# The juvenile systemic sclerosis clinic: an interdisciplinary approach

**DOI:** 10.1186/1546-0096-10-S1-A72

**Published:** 2012-07-13

**Authors:** Marìa Katsicas, Erica Hammermuller, Bettina Cervini, Ricardo Russo

**Affiliations:** 1 Hospital de Pediatrìa Prof Dr JP Garrahan, Caba, Buenos Aires, Argentina

## Purpose

Juvenile Systemic Sclerosis (JSS) is a rare, complex disease. Usually, patients with JSS receive specialized care from a multidisciplinary team in tertiary hospitals. In order to deliver high standard care, systematic clinical practice strategies should be designed. Based on these premises, we developed a JSS Clinic in the year 2008. Objective: To describe the structure and process of a JSS interdisciplinary Clinic, as well as outcomes observed.

## Methods

During the period 2008-2010, patients with a diagnosis of JSS entered a structured follow-up in a dedicated clinic. Disease-specific guidelines and outcome measures were designed and applied. An interdisciplinary team was formed under the leadership of a pediatric rheumatologist: pediatricians, pediatric dermatologists, neurologists, pulmonologists, cardiologists, social workers, nutritionists, kinesiologists , psychiatrists and nurses. The team gathered in the Day Hospital facility of a tertiary pediatric hospital. Prior to the clinic day, short reviews of patients´ charts were distributed electronically. During the clinic day, a specific assessment protocol including several organ systems was applied to all patients. Investigations and outcome measures included: pulmonary function tests and lung imaging, echocardiography, gastrointestinal studies, skin induration (modified Rodnan´s), functional capacity (CHAQ, CMAS), quality of life (QoL) (PedsQL) scores and Padua severity score (Padua SS). After the evaluation phase, all participants held a discussion where tailored strategies were designed for each individual patient. A final report was elaborated and sent to the local chart, data base, and the patient´s primary physician. A proposed set of quality measures for the process of care in JSS was designed and applied. Comparisons between outpatient ordinary care and this interdisciplinary care at the clinic were made.

## Results

Twelve patients (all female) were included in the JSS clinic program. Patients´characteristics: Age at onset 96 (9-180) months; delay in diagnosis 21 (4-48) months. Patients were attended in the clinic every 3 months. The total number of visits to the clinic was 66. Outcome measures (Rodnan, CHAQ, CMAS), were obtained at each visit. QoL and Padua Severity Score (PSS) were assessed once, at the same time. PSS was : moderate= 6 patients ; severe= 4; mild=2. PedsQL Child Self-Report :76,66 (59,72-98,91) and Parent Proxy-Report: 74,45 (40,27-93,48). During the time spent at the clinic, workshops with psychiatrists, parents and patients were held. Measures of quality of care were more frequently present in the JSS clinic program than in outpatient ordinary care (17.5 vs 6.5, p=0,00001).

## Conclusion

The JSS clinic is an interdisciplinary approach that allows a better quality of care, based on the interaction of participating members and the development and use of disease-specific guidelines. A systematic follow-up of patients with a high variability in clinical features and outcomes is best accomplished in this setting.

## Disclosure

Marìa Katsicas: None; Erica Hammermuller: None; Bettina Cervini: None; Ricardo Russo: None.

